# Book Reviews

**Published:** 1830-11

**Authors:** 


### CRITICAL ANALYSES.

Quæ laudanda forent, et quæ culpanda, vicissim

Illa, prius, cretâ; mox hæc, carbone, *notamus*.—Persius.

*The Laws relating to the Medical Profession; with an Account of the Rise and Progress of its various Orders.* By W. Willcock, Esq. Barrister at Law.—8vo. pp. 450. Clarke, Portugal street, London, 1830.

The title of this book is at the present moment especially attractive, and promises amusement, as well as necessary information to the medical reader. Every man should possess, at least, a general knowledge of the laws which govern the community to which he belongs; and yet it is very certain that the majority of medical practitioners know as little of those which preside over their various ranks, as they do of "Coke upon Lyttleton," or the records of the Court of Chancery. This want of information upon the subject cannot fairly be imputed to their carelessness or inattention; for, until the publication of the present volume, the medical practitioner had no legal guide to direct his inquiries: he could neither have time, inclination, nor the necessary talent, to enable him to obtain even a very superficial knowledge of medico-legal subjects, by poring over perplexing charters or legal documents, couched in "quaint and unsavourie speech." A lawyer only could perform this task. It is true that discussions respecting the legal rights and powers of the different medical bodies have recently been much in vogue; but, as they have mostly arisen from party spirit, and have been therefore intemperately and even unfairly conducted, they have rather tended to mislead, than to afford correct information upon the subject.

Mr. Willcock has divided his work into two parts: in the first, he has so treated the subject as to relieve those, to whose pursuits it particularly applies, from the irksomeness of which the general reader has often to complain in the perusal of works more immediately addressed to the lawyer. The second part comprises the Acts of Parliament, the decided cases, and several very curious and ancient records, which will supply the legal student with the information in which the treatise may be found deficient, and the medical reader with the most authentic sources of the history of his profession.

The first chapter, descriptive of the "Ancient Orders of the Medical Profession," we must very briefly refer to. It forms, however, a very necessary introduction to the work, and cannot be perused without amusement.

Passing over the brief, yet interesting, sketch which is given of the first rude dawn of medical science; the doubtful knowledge, mingled with superstitious stratagems of the druids and bards; the total arrest of every science during the Roman and Saxon periods; the description of the monkish practitioners, who perhaps blended with their medical applications a little learning; we proceed at once to the reign of our Eighth Henry, before whose time it appears that we possess but very scanty materials for a history of the medical profession. In the third year of this reign, an Act* was passed, which is generally received as the first operative law on the subject. By this, which is especially aimed against the sorcerers, witches, and smiths, "who can no letters in the book," provision is made both as to sufficiency of learning and purity from supernatural attainments, by appointing the bishop of the diocese to exorcise the fiend, and two doctors to examine the medical qualifications of the candidate. This statute evidently shows that there were many physicians openly and legally practising, who had derived no sanction from Alma Mater; for they alone appear to have been the proper objects of the examination, as the third clause of the same Act provides for the right of the University graduates to practise without undergoing it. In the fourteenth year of the same reign,† another Act was passed relative to physicians only. Their examination was now confided to the College instituted by the charter of the king. Under this, the University graduates, who wished to practise in London, were included, as well as the other physicians; and since that time the legislature has seldom interfered on the subject.

The monkish physicians were at first the only regular surgeons, and they probably sometimes employed the barbers and farriers in the less agreeable part of the occupation. Their brother ecclesiastics became envious of the profits derived from these secular avocations, and at the council of Tours, in 1163, it was declared that none of the regular clergy should devote their attention to physical compositions. This canon is drawn up in respectful terms towards those against whom it was aimed: "It charges them to have been tempted by the arch enemy to undertake duties at variance with their religious character; they are complimented as being the dainty morsels of Satan, the

* P. vi. part ii. † P. vii. part ii.

most worthy members of their profession, and seduced through the best feelings of their nature, a desire to alleviate the sufferings of their brethren." The operative part of surgery being totally abandoned by the scholars, in pursuance of the canon of Tours, it fell into the hands of the barbers and smiths; but the more dignified and regular surgeons were the gentlemen of the Barber's Company. The first recorded instance of the co-operation of physicians and surgeons, is in the unfortunate case of Richard the First, who seems to have been attended and horribly maltreated by both, when wounded at the siege of Chalons: his life was the price of his confidence."

GUIDO DE CAULIACO, who wrote about the middle of the fourteenth century, gives a curious description of the then state of surgery. For example: "the operation of trepanning appears to have been the subject of amusement; and the object of rivalry was to bore as many holes in the wounded skull as the instrument could make, without destroying it altogether." Wonderful virtues are ascribed to the application of a yet bleeding capon to the stump from which a hand or a foot had been severed, after the process of burning.

The apothecaries, or rather the grocers, had their own share in the business long before the distribution of the medical faculty into physicians and surgeons; and it was not until the introduction of chemical medicines, and too great a variety in the mixtures of the learned physicians, had rendered the medical department of their trade unintelligible to the ordinary grocers, that the pharmacopolites appeared as a separate class, and claimed a superiority over the dealers in cheese, butter, and sugars. The grocers, or poticaries, formed one of the ancient companies of the city of London, and it was only in the thirteenth year of James I. that they were formed into two distinct corporations. From a very early period, the apothecaries had occasionally prescribed the medicines which they sold; thus trespassing, as it was thought, on the province of the physician, until their right to do so was supported by the decision of the House of Lords, in the case of the College of Physicians v. Rose; since which they have continued to enjoy this privilege without molestation. So generally had this branch of the faculty superseded, in many instances, the practice of the physician, that, in the fifty-fifth year of the reign of George III., an Act was passed to provide for the sufficiency of their education; thus, for the first time, placing them, as a body, on the footing of a liberal profession.

The empirics were irregular practitioners, who, despising, or ignorant of, the learning of the schoolmen, and neglecting the dogmas of the regular surgeons, chalked out for themselves a peculiar line of practice. Meanwhile the alchymists were occupied in the discovery of the elixir vitæ, the queen of medicine, the philosopher's stone, or by whatever other name the supposed panacea, or rather panourgon, was to be called. "Though the mighty discovery never was, and never will be made, each attempt produced some new result valuable to science, and peculiarly conducive to the progress of medical knowledge." By the astrologers, knowledge was impeded rather than advanced. The most profitable part of their business was thought to consist in discovering the influences of the heavens over the human frame, and especially over the affairs of their particular customers. The sorcerers were another class of the ancient orders of our tribe, and of these there were various descriptions: the arch-sorcerer was the king, who affected to cure the scrofula by the royal touch. The Jewish physicians had learnt the arts of healing practised by the Arabians, Greeks, and other orientals, and introducing them into these parts, without the pomp of learning, were infinitely superior to the leeches of Catholic Europe. In all these sects there were innumerable pretenders.

In the age of chivalry, the high-born damsel deemed it an essential part of her education to acquire some proficiency in the art of healing. Her knowledge was not confined merely to the medicinal virtues of herbs: our ancient poets describe her as occupied in setting broken and dislocated limbs, and superintending the dressing of the wounds of her champion. "The kindness and attention of such a surgeon would, at least, alleviate the mental sufferings of the knight, and probably her simple remedies were, with the aid of nature, the best regimen of the period."

We now approach the consideration of the "Present Orders of the Medical Profession;" a subject in which we must be more immediately interested.

"The law recognises only three orders of the medical profession: physicians, surgeons, and apothecaries. It tolerates, however, such persons as through charity, and without receiving any remuneration, minister to any outward sore, uncome, or apostumation, outward swelling or disease, any herbs, baths, poultices, or emplasters, according to their experience or knowledge; or drinks for the stone, strangury, or agues. Chemists and druggists also are noticed as persons who may make and vend medicines; and it may be difficult to show that they may not compound them according to the prescriptions of a physician, or the orders of an apothecary: but they cannot, in any case, prescribe physic of their own authority." (P. 30.)

PHYSICIANS are, in rank and legal pre-eminence, the first class of medical practitioners. By 32 Henry VIII. they are allowed to practise physic in all its branches, among which surgery is enumerated. The law, therefore, permits them both to prescribe and compound their medicines, and to perform and superintend operations in surgery. These privileges are also reserved to them by the statutes and charters relating to the surgeons and apothecaries. But custom has more decidedly distinguished the classes of the profession. The practice of the physician is universally understood, by their College and the public, to be properly confined to the prescribing of medicines, to be compounded by the apothecaries; and in so far superintending the proceedings of the surgeon, as to aid his operations by prescribing what is necessary to the general health of the patient, and for the purpose of counteracting any internal disease.* English physicians are of two classes: of the College of London, and graduates of Oxford or Cambridge. They are again subdivided into licentiates who may practise within the precinct of London, (that is, in the city, and within seven miles around it,) and licentiates who may practise in other parts of England, and in Wales, and in Berwick upon Tweed, but not within the precinct of London. The licentiates of the College who may practise within the precinct of London are of three orders, fellows, candidates, and mere licentiates; the last are alone generally denominated licentiates.

"The charter (10th Hen. VIII.) granted to J. Chambre, T. Linacre, F. di Victoria, N. Halsewell, J. Francis, and R. Yaxley, that they, and all men of the same faculty of and in the city of London, should be in fact and name one body and perpetual community or college; and that the same community or college might yearly for ever elect and make some prudent man of that community, expert in the faculty of medicine, president of the same college or community, to supervise, observe, and govern, for that year, the said college or community, and all men of the same faculty and their affairs; and also, that the president and college of the said community might elect four every year, who should have the supervision and scrutiny, &c. of all physicians within the precinct of London." (P. 32.)

* R. v. Cowle, 2 Bur. 850-864.

The statute (14 Henry VIII.) confirmed this charter, and further ordained that the six persons above mentioned, choosing to themselves two more of the said commonalty, should from thenceforth be called elects; and that those elects should yearly choose one of them to be president; and then provided for the election of others, to supply the rooms of such elects as should in future be void by death, or otherwise, which was to be made by the survivors of the same elects. By statute 32 Henry VIII., the president, commons, and fellows may elect, at such time as they think fit, four persons of the commons and fellows, to search and examine apothecaries' wares, &c. The college is bound to choose four censors, for the purpose of discharging the duties confided to it.

"It is evident that the charter so far incorporated all persons of the same faculty, of and in London, that every person, on the 23d of September, in the tenth year of the reign of Henry the Eighth, falling within that description, was entitled to be admitted into the association. Such of them as had availed themselves of this privilege, and others subsequently admitted, are the persons described by statute 32 Hen. VIII., as commons and fellows. But as to the persons who should afterwards enjoy that distinction, the original charter, and all subsequent statutes, are silent." (P. 34.)

The effect of 14 Henry VIII. is, that every man possesses his common-law right of practising in the profession of physic as in every other profession, if competent, and by it are appointed the president and college as judges of this competency. They must admit every man, of whose competency in learning, medical knowledge, and integrity they are satisfied, in the exercise of a fair discretion, that they may confide the health of the patient; and every other person they are bound to reject, until he shall have acquired that competency which he does not, on his examination, appear to possess.

"The college has confided the actual examination, as to medical knowledge, to the censors; following the spirit of the statute, which has confided the particular oversight of the faculty to these officers. It has also appointed that the doors of the censor's chamber shall be open to all fellows who may think proper to be present, and that they may take part in the examination should they think fit; and that the fellows may have an opportunity of availing themselves of this right, it is appointed that all examinations shall take place at a court held at certain regular intervals." (P. 41.)

The opinion of the censors is to be ascertained by ballot, and certified to the aggregate meeting of the body. If the censors refuse to examine a person who has applied to them for that purpose, the Court of King's Bench will issue a mandamus to the body at large, requiring them to admit such person to an examination, or to make known some sufficient reason for not doing so.* The reason usually assigned is, that the applicant is deficient in the qualifications which the rules of the college require him to possess previously to his examination.

"The statutes of the college require that every candidate for the licence shall have attained the age of twenty-six years, and have received the degree of doctor of medicine, after two years' residence in some academy for the purpose of studying physic; that every one who has used any nostrum in his practice shall make known its composition and mode of application, to the president or vice-president, and censors; and that every applicant who is a member of the society of surgeons or apothecaries, shall resign his place in such society previously to offering himself for examination." (P. 42.)

The mode of examination is wholly in the discretion of the college. A mandamus would issue against the president and censors, should they refuse to license a person approved of by the Comitia minora, and they must show that they had subsequently examined such person, and found him deficient. The order of candidates was not founded on any Act of Parliament or charter, but was created by the by-laws of the college. They are not, therefore, officers, or even members of the corporation, but mere licentiates, who may, or may not, be afterwards admitted fellows, according to the discretion of the college.

"Although the college has thought proper to prescribe certain qualifications, which it requires those to possess who may seek admission into this order, it does not, nor can it confer on persons possessing that qualification a right, far less an exclusive right, of admission to any distinction beyond that of the licentiates.† All that persons so qualified can claim is, an examination, admission to practise, and the grant of letters testimonial." (P. 44.)

Many and expensive litigations have taken place between the licentiates and fellows, from the claim urged by the former to be considered members of the college. We must refer to the work itself for a detail of the various suits at law, and the different arguments maintained by advocates and judges, upon this often contested point. It appears that the charter incorporated all physicians then legally practising in London, and each of them, who thought proper to accept it, became *ipso facto* a member or fellow;‡ but

* Dr. Letch's case, p. xxix. et seq.

† Dr. Schomberg's case, p. lxxviii.

‡ P. lxxvii.

as all future practitioners, within the precinct of the city, were required to obtain the licence of the college, there soon arose two orders in the profession. The original Act of incorporation gave the fellows an unrestricted right of electing into their number whom they might think proper, "and no person has, or can, by any by-law or regulation of the college, acquire any inchoate right of admission into this order." The fellows have lately admitted several of the licentiates into their association;

"and it may be reasonably expected that the jealously which has for a long time disturbed the harmony of that highly respectable profession, will gradually abate, until at length the manners, virtue, and talents of the man will be deemed in him a higher recommendation to the good opinion of his competitors, than the mere circumstance of his having cultivated these talents within the walls of certain buildings, or on the banks of a certain river." (P. 53.)

The difference between the London and country licentiates consists in the former requiring the consent of a corporate assembly of the college, the latter requiring only the concurrence of the president and three of the elects. Mr. Willcock doubts the legality of the partial licences, which the college were formerly in the habit of granting, to practise in certain complaints only. The only other class of physicians recognised by the law are those who have obtained a degree in physic at Oxford or Cambridge: the lowest degree, that of bachelor, is sufficient to satisfy the terms of the statute.

"In Oxford the degree of bachelor of physic may be now obtained in the thirty-first term, and that of doctor at the expiration of three years from the time of taking the degree of bachelor. In Cambridge, the degree of bachelor cannot be obtained until the candidate has entered his sixth year, and resided nine terms. A doctor of physic must be a bachelor of five years, standing." (P. 56.)

Surgeons. It was once thought that the sole office of the surgeon was to use the knife, and to apply ointments and external medicaments: but this distinction cannot be supported, as many diseases are considered exclusively surgical, for the cure of which internal remedies are necessary; and the surgeon has an undoubted right to prescribe medicine, as auxiliary to the removal of all surgical complaints. The surgeon may make and compound all medicines applicable to the diseases submitted to the superintendence of his branch of the faculty; and he may either administer them himself, or prescribe what he thinks proper to be administered by others.

"There is, in fact, but one class of surgeons, who are legally entitled to practise in this kingdom, the members or licentiates of the college of Surgeons. The statute 3 Hen. VIII. is, however, still in force, though it has been generally regarded as obsolete, inasmuch as there is no instance of any person having obtained a licence under it for several centuries. On this statute alone can any punishment be inflicted on persons for practising surgery without licence in any part of the kingdom, except within London and Westminister, and seven miles around these cities."* (P. 58.)

From an erroneous idea that the original corporation of surgeons was dissolved by the sudden death of the master, on the day of election, a charter was obtained in the 40th year of the reign of Geo. III.*

"As the statute 18 Geo. II. operated to legalise the illegal exclusion of the charter of Charles I., none can practise surgery within London and seven miles around until examined and admitted by the college of surgeons; but there may be two classes of surgeons throughout the rest of the kingdom.

"First, the members of the college of Surgeons, who may practise in every part of his Majesty's dominions; and secondly, the surgeons licensed under the 3 Hen. VIII., who may practise within any particular diocese in which they are licensed, except within London and Westminster, and seven miles around these cities." (P. 64.)

As it has been said of physic, so every man is, by the common law, entitled to practise surgery, if competent. Two classes of judges of that competency have been appointed by statute: the examiners of the Surgeons' company, and the ordinary of the diocese, or, in his absence, the vicar general, with such as he is empowered to associate with him.

"The office of each class of examiners is inquisitorial and judicial. They must perform their duty with impartiality. They must approve every man whom, on examination, they find sufficient: and they will be guilty of a breach of that confidence reposed in them by the legislature, if they approve any one who appears to them to be insufficient." (P. 64.)

The examiners alone have a right to vote for the admission of members. Every person approved of by the majority of the examiners present (and four only are necessary), before two of the governors, is entitled to claim admission. If the examiners refuse to examine a person who has sufficient testimonials of qualification, he may obtain a writ of mandamus, to compel them to do their duty.

"The college has, from time to time, published information as to

* P. clxxxviii.

the testimonials which they require from persons who seek an examination; but these rules are, like those of the college of physicians, directory only, and not obligatory, Inasmuch as they appear to be reasonable, and point out a proper series of studies, the court will be at all times inclined to support them: but it may be stated without qualification, that no body of men to whom a special power is delegated can transfer the exercise of it to others; and that no corporation can by any means impose a qualification on the parties entitled to admission into it, or to an examination preparatory to such admission, which is not imposed by the statute, custom, or charter, on which it is founded. The court of examiners is investigated with a power which they are required to exercise, of examining every person desirous of practising as a surgeon. It may with much propriety inform those who may think proper to present themselves of the nature of the acquirements which they will be expected to possess; but as to the persons and places under whom and in which they may be supposed to have attained them, their rules cannot be obligatory. Should any person to whom an examination had been refused, inform the court of King's Bench that the course of his studies and observations had for a reasonable time been such as to induce a fair presumption that he was competent to sustain an examination by these officers, and that he had informed them of these facts, I doubt not that the writ would be allowed to issue, although it did not appear that he was within the terms of the regulations published by the college. But I doubt much whether such a case is likely to occur, as long as this body is content to adhere to the regulations which they have lately published in this respect.

"Every one desirous of practising in any other part of England than within seven miles around London and Westminister, and who has not been admitted by the college of surgeons, must, before he takes upon him to exercise that science in any diocese within the realm, be examined and approved by the bishop of the same diocese; or, if the bishop be out of the diocese, by his vicar-general; either of them calling to him such expert persons in the faculty as his discretion shall think convenient, and giving his letters testimonial under his seal to him that they shall so approve. If the applicant be approved by the majority of these examiners, he is entitled to claim the proper testimonials, for which, after refusal, the Court of King's Bench would afford him a remedy by issuing its mandamus." (P. 65.)

The proper practice of APOTHECARIES consists in preparing and dispensing medicines prescribed by the legally-recognised physicians, and in applying or administering the same.

"They are also at liberty to administer medicine of their own authority, and without the advice of a physician. It is not usual, however, for them to prescribe medicine to be prepared and supplied by others: but though this is understood to be the peculiar privilege of the physicians, it might be very difficult to make out a case of violating the statute of 14 Hen. VIII. against an apothecary, by merely proving that he had written a prescription, and received such fee as is usually paid to a physician, provided that it did not purport to be the prescription of a physician; and though no person is bound to prepare the medicine prescribed, I am not aware of any penalty incurred by compounding it." (P. 67.)

Two classes of apothecaries are recognised by law: the licentiates of the Apothecaries' company, and a temporary class, such as were in practice on the 1st day of August, 1815. No one can practise as an apothecary in England or Wales, until he has been examined by the court of Examiners of the Apothecaries' company, or the major part of them, and has received their certificate of his being duly qualified to practise as such, unless he were in practice on the 12th day of July, 1815, and also upon the 1st of August, 1815; and no person has a right to claim, or can be admitted to, an examination, until he is twenty-one years of age, and has served an apprenticeship of not less than five years to an apothecary, and produced testimonials of sufficient medical education and of good moral conduct. Non-compliance with these provisions of the Act renders the certificate void; and a false statement of the circumstances required, though not amounting to forgery, even if supported by falsified documents, is a grievous and punishable misdemeanour.

"As evidence of a sufficient medical education, 'to the satisfaction of the court of Examiners,' that court has required the production of certain testimonials mentioned in another part of this work: and although they cannot make rules in this respect wholly unreasonable, they have a far more extensive authority on the subject than the other medical corporations; having the express sanction of an Act of parliament for the exercise of their opinion on the sufficiency of the testimonials." (P. 69.)

If the court of Examiners refuse to examine a qualified person, the Court of King's Bench would compel them to do their duty. No person can act as an assistant to any apothecary in compounding or dispensing medicines, without a certificate of his fitness for that employment from the court of Examiners, or from county examiners, or unless he had acted as an assistant on the 12th July, and the 1st August, 1815, or had then served an apprenticeship of five years to an apothecary.

"The exemption in favor of persons in practice before the 1st August, 1815, extends only to persons who had been in practice as apothecaries; and the statute having in the fourth section described the proper business of apothecaries, none are entitled to the benefit of this exemption who were not, before or on that day, capable of performing such duties. And although the court will not inquire into the degree of skill and capacity of the practitioner in this respect, it will admit evidence showing a total incapacity to perform such duties, as it is conclusive evidence that he had not practised as an apothecary.

"And for the same purpose, although the court will not examine into the extent of practice before or on that day, it will examine whether the practice was in the character and according to the manner of an apothecary; for otherwise every surgeon, dentist, and druggist, and indeed every person who had, either for or without reward, in any manner taken upon him to administer medicine, or to prepare it according to a prescription, would be entitled to practise in all respects as an apothecary." (P. 72.)

Chemists and Druggists. As, in the course of time, the apothecaries have established as a right that which was, at first, considered an encroachment on the department of the physician, the administering of medicine to the sick of their own authority, so the druggists seemed to have acquired a right of compounding physician's prescriptions, which was at first an infringement on the privileges of the apothecaries.

Accoucheurs and Midwives. Any person may act as an accoucheur or midwife, but their acting in this capacity will not warrant their interfering in the cure or relief of any diseases antecedent or consequent upon childbirth.

The administering of medicine gratuitously is not a violation of any law relative to the medical profession.

In the third chapter, Mr. Willcock mentions the various penalties to which unqualified persons are liable, for practising physic or surgery. He is of opinion that an action will lie at the suit of the college of Physicians, although the practice proved be surgical, unless the defendant, by his plea, show that he is legally entitled to practise as a surgeon, by specially setting forth his licence by the college of Surgeons.* The statute 32 Henry VIII. has expressly declared that surgery is a special member of physic, and within the legitimate range of the physician's vocation. Every person, except a physician, is liable to a penalty of five pounds for *every time* he may practise surgery in London or Westminster, or within seven miles of London, unless he has been admitted to practise by the college of surgeons. Every person, except a physician, is liable to a penalty of

* In the case of the College of Physicians v. Harrison, the court held a contrary opinion.

five pounds *for every month* he may practise in any other part of England, unless he has been admitted by the college of Surgeons, or approved of by the ordinary, or, in his absence, the vicar general of his diocese. The Act 55 Geo. III. c. 194, imposes penalties on unqualified persons acting as apothecaries or assistants to apothecaries. In an action for the penalty, the company must prove one act of practice as an apothecary.

"It is necessary that the declaration should allege that the defendant had not obtained the necessary certificate, and that he was not in practice on the 1st of August, 1815. But as these are negative averments, it is not necessary for the plantiff to prove either of them, as it is incumbent on the defendant to prove, either that he has obtained the certificate, or that he was in practice on that day. And it is necessary for him to give reasonable evidence that he was in practice on the 1st August; but this may be fairly inferred from proof that he was in practice a short time before that day, unless the plantiff show that he had discontinued his practice during the interval." (P. 84.)

The defendant's practice must have been as an apothecary; therefore it is insufficient for him to show that he had acted as a druggist, or that he had gratuitously taken upon him to act as an apothecary.

The fourth chapter, entitled "Malpractice in Medicine," defines the various acts coming under that denomination, and describes the power of inflicting punishment for such acts, possessed by the physicians, surgeons, and apothecaries.

The next and brief chapter is concerning the civil responsibility of medical practitioners for want of due skill and attention, and for trying experiments in medicine and surgery. By experiments, Mr. Willcock does not imply the mild and dangerous practices of rash and ignorant practitioners, which come under the head of want of skill and knowledge; but of the deliberate acts of men of considerable knowledge and undoubted talent, differing from those prescribed by the ordinary rules of practice, but which they, in their judgment, believe are likely to benefit the patient. Even in this case, however, the patient must be informed that it is an experiment, and then the practitioner is not answerable in law for the result, although it be contrary to expectation, and attended with an injury which has not generally followed the ordinary mode of practice: but if the practitioner perform such experiment without the knowledge and consent of his patient, he is liable to damages.

Remuneration of Medical Practitioners. It has been recently determined that the fee of a physician is honorary, and that it cannot be recovered by an action at law; and that every person professing to act as a physician is precluded from assuming a different character, as that of a surgeon or apothecary, for the purpose of recovering his fees, although he may, in fact, be a surgeon or apothecary, or a person who had no right to practise as a physician. In the case of Dr. Chorley v. Bolcot, which is related in the second part of the work, it was ruled that a custom, in the defendant's neighbourhood, to pay physicians at a certain rate, is immaterial, and gives them no greater right to bring the action than in places where no such custom is known. It appears that, until the decision in this instance, all the statutes and cases concurred in supporting the right of the physician to maintain an action for his fees.

Surgeons are entitled to recover a reasonable remuneration for their care, attendances, skill, labour, medicines, and applications in surgical cases.

"It has not been required of a surgeon to prove that he is legally entitled to practice, the defendant's having employed the plaintiff in that capacity has been thought sufficient to enable him to support the action, unless the want of qualification is shown. It cannot, however, be necessary to plead this objection especially; for, inasmuch as it is an essential part of the contract that the plantiff should possess the character in which alone he is entitled to recover, the defendant may certainly show, if he can, that he has not been admitted either by the college of Surgeons, or by the ordinary of the diocese. But, inasmuch as this would be attended with great difficulty, it is far more prudent to take the objection in pleading, which, as it is a negative averment, would impose on the plaintiff the necessity of proving his qualification." (P. 113.)

The surgeon is entitled to a compensation for his medicines, as well as for his skill and attention, when such medicines have been applied in cases within his proper province. But if he infringe upon the practice of the physician or apothecary, his conduct being illegal, he has, of course, no remedy for the recovery of any remuneration for his service. A legally-constituted apothecary has the same remedy for the recovery of his fees as a surgeon.

"It may, therefore, be safely laid down that an apothecary may charge for attendances; but that if he do so, he must charge for his medicines at such a rate as to remunerate him merely for their intrinsic value, and his skill and trouble in compounding, and to exclude any compensation for his attending to apply or administer them." (P. 118.)

Towne v. Gresley, and Hanley v. Henson, are both cases which confirm this opinion.

Protection of Medical Men. In cases of defamation or libel, a regular practitioner is not bound to prove that he has sustained actual injury or loss; the law will presume that injury must arise from the propagation of such calumny. The law will not require the plaintiff, in an action for libel or slander, to prove the falsehood of the charge against him; it will allow the defendant to prove the truth of the imputation which is charged to be libellous or slanderous, provided that he has given due notice, so that the plaintiff may have an opportunity of producing his evidence to refute or explain it.

"Another ground of defence against an action for libel or slander is, that the imputation was not malicious; and this is admissible as well on an indictment as on a civil proceeding. For, though the law, to prevent the excitement and disturbances arising from defamatory imputations, however true, will not in general admit the truth of the charge to be pleaded to an indictment which is supposed to be the prosecution of the king, and founded upon the necessity of securing the quiet and peace of the nation, it will permit the defendant even on an indictment to produce any evidence tending to show that the imputation was not in legal acceptation malicious." (P. 121.)

If the defendant can satisfy the jury that the alleged libel or slander was not malicious, he is entitled to a verdict in his favor. Confidential communications are not slanderous. If, therefore, it appear that the calumny was contained in a letter written, or words spoken in confidence, to a person who had consulted the defendant with relation to an intention to apply to the plaintiff professionally, or to a person so related to, or connected with, the defendant, as to justify his dissuading him from consulting the plaintiff as a professional man, it is merely necessary for the defendant to show that he conscientiously believed the imputations to be just, and such as a prudent man would use for that purpose. But

"a confidential communication defamatory of another is legal only to the extent in which it is justified by an intention to benefit the person to whom it is made; and if abused for the purpose of injuring the person calumniated, is more severely to be punished than public defamation, inasmuch as it deprives the accused of the opportunity of being heard in his defence, which is afforded him by making the charge public." (P. 122.)

Fair criticism, however severe or calumnious, is not punishable as malevolent slander; but it must be confined to its proper subjects, public acts and public writings. The critic is not bound to substantiate the correctness of his observations, but he is liable to punishment for either unfairly stating the facts which he pretends to criticise, or for introducing private character as the subject of improper remarks. The cases of Macleod v. Wakley, Cooper v. Wakley, and Dunne v. Anderson, are to this point.

The privileges of medical men in respect of insane persons, in respect of employment, exemption from serving on juries and in offices, and from bearing arms, form the subjects of the eighth chapter.

The law relating to the propagation of contagious diseases, and other injuries to the public health, are next considered. "Any unnecessary exposure of persons infected with smallpox, is a nuisance punishable at common law, whether such persons become infected by communication with others, or their disease originated in inoculation."

The first part of the work concludes with some interesting remarks "on Dissection." Any medical man, or other person, concealing the fact of a body having upon it such *indicia* of violence, or receiving a body under such circumstances as to raise a reasonable suspicion that the person had been wilfully destroyed, is regarded by the law as an accomplice in the crime.

We can only name the contents of the second part of the volume. The various statutes, charters, by-laws, &c. relative to physicians, surgeons, and apothecaries, are given at length; and many amusing and very instructive actions at law are reported upon different subjects connected with the medical profession, which cannot but be important and interesting to those of our brethren who may be forced into the thorny path of litigation, either as individuals or corporate bodies, inasmuch as they embrace the opinions of judges, arguments of counsel, and decisions of juries, upon most professional points that are likely to become the subjects of dispute.

Some valuable parts of this work we have been compelled to pass over, but we have drawn enough from the information it contains to convince every medical practitioner, that this is one of the books which his curiosity and interests will lead him to peruse; and that in Mr. Willcock he will find an able and intelligent legal adviser. The College of Physicians would probably not have had to lament their mortifying defeat in their contest with Dr. Harrison, if the argument which Mr. W. acutely suggests had been urged upon the trial. 438.

*Practical Observations on Leucorrhœa, Fluor Albus, or "Weakness:" with Cases illustrative of a new Mode of Treatment.* By George Jewel, Member of the Royal College of Surgeons; one of the Accoucheurs of the St. George's and St. James's Dispensary, and to the Middlesex General Dispensary; Lecturer on Midwifery and the Diseases of Women and Children, &c.—8vo. pp. 108. Wilson, 1830.

Although leucorrhœa is one of those diseases which very often does not materially affect the health of the patient, and perhaps never endangers life, it is still, from its difficulty of cure, an *opprobrium medicorum.* The profession must therefore feel much indebted to any practitioner that is fortunate enough to hit upon a more efficacious remedy than we yet possess for a complaint which perplexes the physician, and annoys the patient by its obstinate continuance. The chief motive which Mr. Jewel has in view in the publication of the present volume, is to suggest a remedy which he has found remarkably successful. The pathological view which he takes of vaginal discharges he believes to be "somewhat novel;" but it will be found, upon reference to Dr. Dewees,* that very similar notions have been before entertained. The principal feature in the treatment here recommended is, we believe, entirely new. Mr. Jewel very properly laments that the opinion too widely prevails that vaginal discharges have their origin in constitutional or local debility, and that hence a complaint of this kind is denominated a "weakness." The employment of this term leads to practical error; for, if we investigate the pathology of leucorrhœal discharges, we shall find that they most commonly have their origin in local excitement. To this part of the subject the author has especially attended, and he believes that the vaginal discharge is commonly the result of some direct local stimulus. Dr. Dewees believes that leucorrhœa has also invariably a local origin. In this opinion our own experience leads us to concur.

Much diversity of opinion exists among writers as to the precise structure which is primarily affected in leucorrhœa, and which may be considered as the seat of the disease. Mr. Jewel thinks that the discharge seldom issues from the uterine cavity; and here again he is supported by Dr.

* Dewees on the Diseases of Females, chap. xiii.

Dewees, who states, in the work to which we have just referred, "We are of opinion that this discharge rarely proceeds from the cavity of the uterus, not even in its most aggravated forms; and when it does, it must always be looked upon as the most difficult of management." (P. 229.) It has been proved by Bichat and others, that the uterus is lined with a mucous membrane, and like other mucous membranes, when in a state of morbid action, it may occasionally throw out a superabundant quantity of mucus, or even pus.

"By attending to the following circumstances, we shall, in some cases, be able to ascertain whether the discharge issues from the uterine cavity or not. When the seat of the disease is in the vagina, or cervix of the uterus, the discharge commonly appears during the night, notwithstanding the patient is confined to the horizontal position; but, if in the cavity of the womb, it is generally suspended: a piece of sponge, therefore, being introduced into the vagina at bedtime, will occasionally determine the question; for if the discharge issues from the surface of the vagina, it will become saturated with it." (P. 9.)

We give this passage from Mr. Jewel, but he should himself have inserted the inverted commas, as it is taken almost literally from Dewees, p. 234.*

In investigating the pathology of leucorrhœa, there are three states or conditions to be regarded, says Mr. Jewel, as being almost invariably the immediate causes of the disease in question: namely, irritation, congestion, and inflammation of the subacute or chronic kind. We question whether the distinction which is here presumed to exist between a state of "irritation" and one of "sub-acute inflammation" in leucorrhœa, can ever serve us in practice, or be even so satisfactorily described by words, as to induce us to believe that it is not more imaginary than real.

"From a strict pathological investigation into the numerous cases of leucorrhœa which have fallen under my observation, I have been induced to believe that, when the morbid secretion is abundant, and the local symptoms severe, one uterine affection gives rise to the disease more frequently than any other, namely, a subacute or chronic inflammation of the cervix uteri. Even when the vaginal surface appears to have been the tissue primarily affected, we shall find, in almost all protracted cases, the cervix uteri also seriously involved in the mischief. Mr. Burns, an authority which cannot be quoted without respect, alludes to this part of the subject by observing 'that, when the discharge is very opaque, and attended by considerable pain in the back and loins,

* We refer to the Philadelphia edition of Dr. Dewees' work.

there is reason to think that the cervix uteri is in a state of irritation; and by examination may be found tender to the touch, the mouth soft and enlarged a little. This state does not constitute disease of structure, though it may lead to it, but it consists merely in an affection of the glands. After the tender state is nearly subdued, and the discharge has become more chronic, the cold bath, tonics, and astringent injections are proper.' Here it is evidently meant that, as long as the tender state of the cervix uteri continues, we ought not to employ those means which are usually had recourse to with a view of giving tone and vigour to the system, which, in fact, is an admission of the inflammatory character of the disease. I have scarcely ever seen an instance of profuse leucorrhœa without more or less tenderness of the cervix uteri. I have also reason to know that very many of such cases are mistaken for carcinoma uteri, and that, in consequence, no remedies are prescribed, or a very inefficient mode of practice is adopted." (P. 24.)

Mr. Jewel has seen more than one case where a morbid affection of the cervix uteri had been pronounced by eminent practitioners to be carcinoma, but in which the disease had been afterwards totally eradicated, the uterus again taking on its healthy functions, and the woman bearing children as before.

"To practitioners unaccustomed to examining into the state of the os and cervix uteri, it may not be irrelevant to observe that the form of the os uteri occasionally varies. In some females, instead of its being a small transverse opening, it will be found of a circular form, and now and then so small as scarcely to admit the point of a probe, whilst in others, who have had many children, the point of the finger can be easily introduced; and instead of being uniform, and smooth or flat, it not unfrequently becomes projecting, having an anterior and posterior lip." (P. 32.)

We are very judiciously warned not too hastily to consider scirrhus an incurable disease, or that it so exists throughout the system as to become developed in one part when eradicated from another. Dr. Denman, when speaking of cancer in the uterus and breasts, says, when the former is the seat of the disease, the first symptom is usually inflammation; and, if this can be effectually removed, by strict abstinence, by bleeding occasionally, by antiphlogistic medicines, and by constant repose in the horizontal position, he has often persuaded himself that the disease has been prevented or removed. Dr. Clarke also observes that, by a strict attention to management, and an unwearied perseverance in the means suggested, all cases of the complaint may be relieved; in many, the further enlargement of the tumor, or progress of the thickening, may be prevented; and, if he were not afraid of deceiving himself, or of deceiving others, he would venture to express a belief that, in a few instances, the disease has altogether subsided. Mr. Jewel allows that other judicious and active measures may control such affections, whether they happen to be genuine cases of incipient scirrhus, chronic inflammation, irritation, or simple enlargement from congestion; but he assures us that there is no remedy which can be employed with a greater prospect of success than the nitrate of silver, applied immediately to the part affected. As the principal purpose of this work is to point out the manner in which this remedy is to be employed in conjunction with others, we shall pass on at once to the sixth chapter, in which the treatment of leucorrhœa is considered.

Although Mr. Jewel places the most perfect reliance in the nitrate of silver, he would not exclude what are termed general principles of treatment. If a female labours under leucorrhœa who has a full pulse, determination to the cerebral vessels, thirst, coated tongue, and other symptoms of excitement or fever, bleeding, purgation, and a vegetable diet may remove the complaint, without the employment of particular remedies. Until these plethoric symptoms are removed, local remedies would be injudicious, with the exception of local ablutions. Water alone for this purpose is said to be "very nearly, if not totally, a useless practice. If, however, one part of vinegar be added to two parts of water, and the whole made lukewarm, it will be found a useful and an agreeable auxiliary." We know no objection to the addition of the vinegar, but we are quite certain that the advantages arising from ablution with simple water are here much underrated.

We believe Mr. Jewel is correct in stating that, although general bloodletting will diminish arterial action, it seldom affords relief to the patient under the ordinary circumstances of vaginal discharge. Cupping glasses should be applied to the loins, or leeches to the groins. If the constitution has suffered materially from a long continuance of the disease, we should not take away more than four or six ounces of blood, if, indeed, bleeding be admissible at all in such cases. To relieve the utero-vaginal pains, and procure rest, hyoscyamus or conium are to be given.

"In all leucorrhœal or uterine affections, whether of the acute or chronic kind, absolute rest of the body is indispensable. The patient should remain, during the greater part of the day, in the horizontal position, either on a mattress or sofa; but it must be observed, that, when there is a great disturbance in the nervous system, a moderate degree of passive exercise, such as riding in a carriage, will prove highly beneficial in its tranquillization, and in restoring the functions and general health." (P. 70.)

This passage appears to us to be incautiously worded; for we can scarcely believe that Mr. Jewel seriously means to maintain the doctrine included in it. It would be absurd to attempt to confine "*all*" patients labouring under leucorrhœa, in a state of absolute rest; and if they would submit to so unnecessary an injunction, we could not with propriety enforce it. In the great majority of cases, moderate exercise is undoubtedly necessary, and such a degree of it may be safely enjoyed as will benefit the general health of the patient, without at all aggravating her local complaint.

Purgatives are generally required in the treatment of leucorrhœa. Cold general bathing is a popular remedy, where a loss of tone in the system exists; but Mr. Jewel judiciously observes, that when the female is much enfeebled by the discharge, where great fatigue is induced by slight exertion, and where she breathes with difficulty, the cold bath must be used with caution, particularly if the secretions of the digestive organs have become obstructed. In cases of acute inflammation of the cervix uteri, the hip-bath, of a temperature from ninety to ninety-five degrees, will generally relieve. Cantharides is sometimes a very decisive remedy in leucorrhœa, but, "notwithstanding a train of symptoms of a very distressing nature may be produced by its exhibition, it sometimes exerts no control over the disease in question. Within the last six months I have seen two cases, in which strangury had been induced without any good result." Of this remedy Dr. Dewees thinks very highly; but the tincture of cantharides he employs is fifty per cent. stronger than the ordinary tincture of the shops.* It is notorious that but little dependence can be placed upon the various local astringents that are in common use in cases of leucorrhœa. Iodine has as yet been but little employed in this disease, but Mr. Jewel states that, in almost all the cases in which he has employed it, its effects have been marked and decisive.

After briefly mentioning the treatment commonly had recourse to in leucorrhœa, and adverting to those general principles, the observance of which is essential to the suc-

* Dewees, *loco citato*, p. 240.

cess of the remedy Mr. Jewel especially relies upon, he observes, that

"In the application of the nitrate of silver to the surface of parts, in a morbid or unhealthy state, a most obvious change is almost immediately produced, which (although we are incapable of explaining it philosophically,) eventually terminates in healthy action. One circumstance, more particularly, led me to adopt the use of the nitrate of silver in the cure of these diseases, namely, the extensive and healthy changes which I have known to result, from the application of this agent to the different mucous tissues, when their secreting surfaces had taken on a disordered or unhealthy action, as in those of the fauces and larynx. After extensive trials and observation, I can confidently say, that its effects are as conspicuous in cases of vaginal discharge, not dependent on disorganized structure, as in the various local diseases in which it has hitherto been employed, with so much success. It has been said, that checking the vaginal discharge is prejudicial: this opinion is at variance with my own experience; but I would employ the nitrate of silver, not merely with a view of arresting the discharge, but to produce a perfectly new action, or new excitement, in the part from which the secretion has its origin.

"The mode I have adopted in the application of this agent, has been either to conceal it in a silver tube, as it is employed in cases of stricture, (except that the tube should be adapted to the size of the argent. nitrat.) or in the form of solution, in the proportion generally of three grains to the ounce of distilled water, the strength being gradually increased. A piece of soft lint may be moistened with the solution, and introduced, for a short period, into the vagina several times in the day; or a bit of sponge, firmly and neatly tied to the end of a slip of whalebone, may be passed into the vagina, up to the os and cervix uteri, well saturated with the solution. This can easily be effected by the patient herself. It is necessary that the application should be frequently repeated, or no permanent benefit can be expected. Should it become requisite to employ a strong solution, and to apply it to a certain part, or ulcerated surface, it can be accomplished with a degree of nicety, by means of a camel's hair brush, introduced through the speculum, or dilator. This, however, can only be done in the absence of excoriations, or tenderness, as the introduction even of a common syringe sometimes produces a considerable degree of pain and irritation; independently of which, some females will not submit to the introduction of any instrument. In married women, there is not the least difficulty in using the dilator, neither does its introduction, under common circumstances, occasion any degree of pain. By means of this instrument, the condition of the cervix uteri and vagina can be readily ascertained.

"A few remarks upon the use and choice of the syringe, when injections are employed. will not, I trust, be considered a digression. It must be obvious, that if the act of throwing in the injection be attended by any muscular effort, the injected fluid cannot reach its destined point, namely, the neck of the womb, and upper part of the vagina. In using the common straight syringe, a degree of bodily exertion cannot be avoided, whatever may be the position of the patient, and consequently the operation must prove very inefficient, if not altogether useless. The pipe of the syringe ought to be curved, so that, when introduced, its point may come in immediate apposition to the os uteri, and the patient should place herself in the recumbent posture, in which position she should remain at least several minutes after the syringe has been withdrawn. The principal advantage in injecting the fluid is, that if any superficial ulcerations exist, they will be readily healed.

"It is very satisfactory to observe, that the nitrate of silver, when judiciously used, in either of the forms above recommended, gives no pain nor irritation, at least no more than is occasionally produced by the injection of any common astringent." (P. 81.)

Several cases are detailed, which appear to show the good effects of the plan recommended. Mr. Jewel has also tried the same remedy in cases of gonorrhœa in the female, with much success; and has found that it may be employed, with perfect safety and advantage, during the presence of inflammatory symptoms.

---

*A Treatise on the Venereal Diseases of the Eye.* By WILLIAM LAWRENCE, F.R.S.; late Professor of Anatomy and Surgery to the Royal College of Surgeons in London; Surgeon to St. Bartholomew's Hospital, and Lecturer on Surgery at that Hospital; Surgeon to Bridewell and Bethlehem Hospitals; consulting Surgeon to the London Fever Hospital, and late Surgeon to the London Ophthalmic Infirmary, &c.—8vo. pp. 337. Wilson, 1830.

(Continued from p. 342.)

BEFORE we proceed to the consideration of the second part of Mr. LAWRENCE's work, we must for a moment refer to the subject of our first analysis, for the purpose of adducing additional evidence in support of our opinion of the superiority of the stimulant plan of treatment over the antiphlogistic in gonorrhœal ophthalmia. In the last Number of the Edinburgh Med. and Surg. Journal, the reviewer of Mr. L.'s work states, that he has been informed by Dr. SHORTT, who had occasion to treat the purulent ophthalmia, on the most extensive scale, in the military hospitals of Egypt and Sicily, that he carried personally the depleting system of treatment to the greatest extent, to 60, 70, 100, and even 200 ounces, and often without the smallest effect in arresting the progress, or mitigating the severity of the disease. In most of the cases the cornea gave way, and allowed, the iris to protrude, where there was no want either of energy, of promptitude, or of extent, in the use of the antiphlogistic measures. The reviewer says that, "pathologically and therapeutically, gonorrhœal ophthalmia is quite the same as the puriform epidemic disease, and the remedies which are beneficial in the latter promise also success in the former." We are gratified to find our highly respectable contemporary agree with us in opinion upon this point.* Dr. Shortt also remarked that those patients who underwent the severest depletory measures were invariably longer in recovering sight, than those treated with more moderation; and in such subjects it was only when the constitutional powers began to rally, that the specks and opacities of the cornea began to disappear, and the ulcerations to heal. These unfavorable results led Dr. Shortt, with others, to employ the nitrate of silver in solution, with tonics and stimulants; and since the adoption of this practice, he has effected a much larger proportion of cures.

We now arrive at that part of Mr. Lawrence's work which is dedicated to "Syphilitic Diseases of the Eye." Under this head, *syphilitic iritis*, and *syphilitic ulceration of the eyelids*, are considered. The iris is liable to inflammation from various causes, of which syphilitic infection is, perhaps, the most frequent. The several forms of the complaint are alike in essential characters; they differ only in modifications of the phenomena, and in concomitant circumstances. Hence a description of the origin, progress, and various effects of syphilitic iritis will embrace nearly all the points that could be included in a general history of the affection.

*General character of Iritis.* The chambers of the aqueous humor, having a smooth membranous lining, which exhales and absorbs a watery fluid, present a striking analogy to the serous cavities. A similar correspondence is observed in their diseases; inflammation being generally attended in the former, as it is in the serous membranes, by the effusion either of albuminous fluid or of coagulating lymph. The peculiar structure and the situation of the affected part will, perhaps, account satisfactorily for our not being able to recognise in iritis the four circumstances, the combination of which is usually considered to constitute the state of inflammation, viz. swelling, redness, heat, and pain. The inflammatory process, however, is unequivocally characterised by one of its effects; that is, by effusion from the vessels of the part. The new matter thus produced,

* Vide our last Number, p. 342.

which is found under various circumstances and appearances, is called indiscriminately by the not very precise term of *coagulable lymph.* Besides changing the colour and general appearance of the iris, it impairs and destroys its motions, rendering it at first sluggish, and afterwards motionless; it causes adhesion of the iris to the surrounding parts; it alters the form and size of the pupil, contracting or entirely obstructing that aperture, with more or less injury to, or complete loss of sight. Thus iritis belongs to the class of adhesive inflammations. The increased action in the vessels of the part, by which the changes just enumerated are produced, is attended by an enlargement of the vascular trunks and ramifications in the sclerotic coat, and consequently by preternatural redness of the eye, to which are usually added increased sensibility to light, and lachrymal discharge.

*Change of colour in the Iris.*

"The change of colour which the organ undergoes, is one of the most striking characters of iritis. A light coloured iris assumes, under inflammation, a yellowish or greenish tint; occasionally, it is distinctly yellow; and, if the eye be blue, a bright green is sometimes seen. Generally, however, the tint, whether yellow or green, is of a dull and muddy cast, and darker than in the sound state. In case of the iris being naturally dark-coloured, it presents, when inflamed, a reddish tinge. Together with these changes of colour, there is a complete loss of its natural brilliancy; it becomes dull and dark, and the beautiful fibrous arrangement, which characterises it in the healthy state, is either confused or entirely lost. These changes, which are rendered particularly obvious by the contrast between the inflamed and the sound eye, commence in the pupillary margin. In an early period, the very edge of the pupil alone may be affected; the internal circle then becomes altered in colour, and thickened; and afterwards the change spreads gradually to the external or ciliary edge of the iris. This alteration of colour is produced by effusion into the texture of the organ; and the particular tint is such as would arise from blending with the natural colour of the iris that of the lymph, which is yellowish or brownish." (P. 132.)

*Effusion of Lymph; its various appearances.* The deposition of lymph takes place under various modifications in syphilitic iritis. The effusion into the texture of the iris generally causes the changes of colour just described. It may be deposited in a thin layer, covering a larger or smaller surface. In different-coloured irides, different appearances are produced by the effusion of lymph. When the inflammation is very active, and has been neglected or improperly treated, the lymph is sometimes secreted so abundantly as nearly to fill the anterior chamber. Under violent inflammatory action, blood itself is sometimes effused. Lymph may be poured out from the margin of the pupil, or the uvea, so as to agglutinate them partially or generally to the capsule of the crystalline. A mass of lymph sometimes fills the pupil.

"More commonly, a thin greyish web or film stretches across the opening, which loses its clear black colour, and has a cloudy appearance. Lymph may be effused in considerable quantity into the posterior chamber, and either make its way through the pupil into the anterior chamber, cause a bulging of the sclerotica, or penetrate that membrane, and form a tumor under the conjunctiva." (P. 135.)

"The external swelling in these cases has sometimes a yellowish appearance on its most prominent part, from which, in conjunction with the intense redness and violent pain of the eye, it has been supposed that suppuration of the globe had occurred; and the part has been punctured, under that notion. I once did this in the case of a lady, whose eye was destroyed by syphilitic inflammation: neither matter nor lymph escaped from the opening." (P. 137.)

Mr. Lawrence believes that suppuration never takes place in syphilitic iritis; that the inflammation, however violent, is always of the adhesive kind, and that the changes to which it leads are produced by the effusion of lymph.

*Motions of the iris, and state of the pupil.*

"The motions of the iris must be seriously impaired by the changes just described, more particularly by the interstitial effusion of lymph. It moves sluggishly at the commencement of the inflammation; and, when effusion has taken place, its movements are entirely suspended, the preternatural connexions by adhesion concurring with the change of structure in producing this effect. The pupil, consequently, cannot exhibit the ordinary variations in size; it is contracted, and it becomes smaller and smaller in the progress of the affection. At the same time the effusions of lymph and the adhesions change the figure of the opening, rendering it angular, and often extremely irregular. Together with other changes, the pupil sometimes undergoes an alteration in situation, being apparently drawn upwards and inwards, or towards the root of the nose. It may deviate in other directions. The margin of the aperture is thickened, and has a villous or spongy appearance in the beginning of the disease, presenting a strong contrast to the thin, sharp, and well defined edge which naturally belongs to it. The effusion of lymph into the aperture, which has been already noticed, destroys its clear black colour, and gives it a dull, cloudy appearance." (P. 141.)

*Increased Redness of the Eye.* In the commencement of the affection, the anterior part of the sclerotica exhibits a pale pink redness, and the vascular trunks, which lie on this membrane, are seen of a deeper pink tint under the conjunctiva, which is then unaltered. As the affection proceeds, the redness of the sclerotica, and the distention of its trunks, increase. In iritis of the most violent kind, all the external vessels of the globe are equally affected, giving to the entire surface an uniform fiery redness. Either a part or the whole of the iris may be inflamed.

*State of the Cornea and Aqueous Humor.*

"The phenomena of the disease shew that an intimate vascular connexion exists between the sclerotica and the cornea and iris, although we do not know much about the arrangements of their vessels in the healthy state of the organ. Hence it happens that active inflammation in either of the latter parts causes vascular distention and redness of the sclerotic, while inflammation originating in that membrane soon extends to them. Hence, when the sclerotica is inflamed, as it is in an acute attack of iritis, change may be anticipated in the state of the cornea. General haziness occurs at first; this is aggravated, as the case proceeds, and nebulous opacity comes on when the inflammation is violent and long continued. This change affects the cornea generally, in most cases; there may be more considerable partial opacity with the general haziness or nebula. Sometimes, but rarely, there is ulceration of the cornea. These corneal affections add to the imperfection of sight caused by the changes in the pupil. Under the existence of inflammation in the surfaces which secrete the aqueous humor, we might expect that this fluid would be altered in its properties, and become turbid. We have, however, no clear evidence on this point. In considerable and active inflammation of the iris, with the cornea remaining clear, we can see no change in the aqueous fluid: when the cornea becomes hazy or opaque, we can hardly expect to discern it." (P. 144.)

Intolerance of light and pain are, as might be expected, concomitant with iritis. The constitutional disturbance is very various. Iritis of the most acute kind is attended with severe febrile symptoms; with headach, restlessness, and want of sleep; with full and strong pulse, white tongue, thirst, loss of appetite, and costiveness. It often happens, however, even in cases that would be termed acute, such symptoms exist only in a slight degree, (Case xix.) or are entirely wanting, (Cases xii. and xx.) When iritis has attained its full developement, and continues, the iris swells, or appears to swell; that is, it approaches towards the cornea, becoming convex in front, diminishing the anterior chamber, and sometimes having its surface puckered and irregular. "Is this an actual swelling of the iris, real thickening of the part from interstitial deposition? or mere protrusion by the swelling of parts behind, by the effusion of lymph, or by aqueous secretion? Dissection has not yet elucidated these questions." If the progress of the affection be not checked, it does not remain limited to its original seat in the iris. Supposing it to go on without interruption, it passes from the ciliary circumference of the iris to the corpus ciliare, the choroid coat and retina, with increase of pain and fever, and ultimately with irrecoverable loss of vision, from change of structure in the retina. Ultimately simple iritis may become ophthalmitis, or inflammation involving the external and internal tunics generally. "The question naturally occurs, whether the inflammation, when thus propagated to the posterior tunics, presents in them the same characters as in its original seat; that is, whether it is attended by effusion of lymph?" Mr. Lawrence has never had an opportunity of dissecting an eye in this state of disease, nor are any such dissections recorded. Iritis, like other inflammations, varies in rate of progress and degree. Serious mischief may occur in a few days, or weeks may elapse without any permanent change of structure or injury of sight. Many of the cases detailed by Mr. Lawrence exemplify these facts.

*Effects of Iritis.*

"The effusion into the texture, or on the surface of the iris, like the interstitial deposition which produces swelling of other inflamed parts, is removed by absorption when the inflammation is at an end. Under favorable circumstances, that is, when the inflammation is recent, and proper treatment has been adopted, the iris may be completely restored, recovering its natural colour, brilliancy, and power of motion. This restoration may take place equally whether the organ should have been simply discoloured, or effusion of lymph should have taken plaee, either in a thin stratum or in tubercular masses." (P. 153.)

Adhesions of the pupil to the crystalline capsule often occur. From proper treatment, in an early stage, the slender threads of adhesion between these parts are sometimes detached, leaving behind (at least in some instances) black marks in the capsule, which Mr. L. believes are permanent. A tubercle of lymph effused on the edge of the pupil will produce a broader adhesion, fixing, perhaps, one third or one fourth of the circle. These changes must necessarily affect the figure and motions of the pupil; they often render it very irregular, and impair or destroy its motions. "Mere alterations of figure are not injurious to vision; which is just as good with the most irregularly-shaped pupil as with a circular one; and we often see perfect vision with great and permanent contraction of this aperture." It must be understood, of course, that the retina is uninjured, and that the pupil, however irregular or small, is clear. When considerable general effusion has taken place, and has been allowedto proceed uncontrouled for some weeks, permanent change is produced in the texture and colour of the iris. If the lymph thrown out into the pupil, and lying on the crystalline capsule, be not soon absorbed, it becomes organised, and forms an opaque adventitious membrane, adherent to the capsule and to the pupil.

"The opacity of this new production is greatest in the centre, and gradually shaded off towards the circumference. In the contracted state of the pupil, it fills the whole aperture; but, when the edge of the iris is withdrawn, it is surrounded, partially or entirely, by a clear black margin, and the iris is found to be attached to it by adhesions, which may be either close or in the form of short black threads. These adhesions sometimes divide the clear portion of the pupil into small roundish or irregular apertures. In such cases, the pupil does not change under variations in the quantity of light; it is usually necessary to apply belladonna in order to expose the clear part of the opening, and the adhesions which connect its margin to the adventitious membrane. If the effusion should have occupied the pupil and margin of the iris only partially, the adventitious membrane will be found towards one side instead of the centre, the edge of the pupil being fixed to it, and thus drawn out of its regular line, while the rest of the opening is natural. This state, which has been called imperfect closure of the pupil (*atresia iridis imperfecta*,) is attended with greater or less injury of sight. Although the patient may have no useful vision when the aperture is contracted, he may be able even to read if a little enlargement can be procured by the influence of belladonna." (P. 156.)

Complete closure of the pupil (*atresia iridis perfecta*) may occur; the communication between the two chambers is then destroyed, and the passage of light into the eye almost entirely intercepted, with corresponding loss of sight. When the inflammation has extended to the posterior tunics, although it should have been arrested by proper treatment, various degrees of impaired vision remain. After the apparent cure of the disorder, the eye sometimes remains preternaturally sensible to external influences.

"*Diagnosis.* The tubercular depositions of lymph, the reddish brown discoloration of the iris on its inner circle, the nocturnal exacerbations of pain, which is felt either in a much slighter degree or not at all during the day, the angular disfiguration of the pupil, and its displacement towards the root of the nose, together with the previous occurrence of syphilis, and, in most instances, the concomitant existence of other syphilitic symptoms, clearly designate this kind of iritis and distinguish it from other forms of the affection. The local symptoms alone are not sufficient, in all cases, to establish the distinction; for we sometimes see merely a general discoloration of the part, such as might occur in idiopathic or arthritic iritis." (P. 160.)

Both in the idiopathic and arthritic iritis, the pupil generally retains its circular figure and central position in the iris.

*Causes.* Syphilitic iritis must be viewed in the same light as other secondary symptoms. The only reason to be assigned for its occurrence is the previous existence of primary syphilis; or, to use the ordinary language, the contamination of the body by the venereal poison. It may happen that cold, wet, &c. will excite the complaint in those already disposed to it, by having previously contracted syphilis; but it appears, in most instances, without any assignable external cause. Syphilitic iritis may occur alone,* but it is more commonly accompanied by other secondary symptoms. It may show itself before the primary disorder is cured.† Iritis is rarely seen as a symptom of syphilis in infants.

An opinion has partially prevailed that the use of mercury is capable of producing iritis. "I have seen no instance of iritis, of whatever kind, in which there has appeared to me any reason for ascribing the occurrence of the complaint to this cause." Iritis occurred in some cases of syphilis which had been treated by Mr. Rose‡ and Dr. J. Thomson|| without mercury. Dr. Ekstrom, of Stockholm, informed Mr. Lawrence that he had seen many similar instances where the use of mercury in syphilis had been entirely abandoned for a long time.

The *prognosis* is favorable when the affection is recent, and confined to its original seat in the iris. We need not fear for the result, if the changes, however considerable, are confined to the iris. The mere quantity of effusion is of little moment. Before pronouncing our prognosis, we should closely examine the organ, in order to decide whether the posterior tunics are involved.

"The state of vision alone will not determine the point: the changes in the cornea and pupil may impair sight considerably, so

* Cases 3, 6, 7, 8, 9, 10, 13, detailed by Mr. Lawrence.

† Cases 5, 20, 25, 26, 27.

‡ Med.-Chir. Trans. vol. iii. p. 361, and Cases in Mr. Lawrence's work, 17, 18, and 19.

|| Edinburgh Med. and Surg. Journal, vol. xiv. p. 91.

that the patient may be unable to distinguish objects, and may be reduced to the mere power of discriminating light and darkness in cases where the function of the organ is ultimately restored. Indeed, a considerably impaired state of vision is sometimes found where the cornea is clear, and the pupil not visibly obstructed, and yet the sense is recovered; so that even affection of the retina is not necessarily a ground of unfavorable prognosis. The case is hopeless when we find a change of colour in the whole iris, with considerable contraction of the pupil and an opaque substance in it, with intense external redness, great and deep-seated pain, and complete extinction of sight. I have not seen vision recovered when large effusion has taken place behind the iris, more particularly if it should have caused bulging of the sclerotica, or have made its way through that membrane. Great contraction and general adhesion of the pupil, a protruded and puckered state of the iris, are very unfavorable circumstances. Considerable imperfection of sight may be removed if the inflammation be recent, but not if it be of long standing." (P. 167.)

It is necessary to take a combined view of the activity and duration of the inflammation before we decide on the probable termination. Much good is often accomplished in cases that seem desperate.

*Treatment.* Three principal objects are to be had in view: to arrest inflammation, to prevent further effusion of lymph, and promote the absorption of that already poured out, and to prevent contraction of the pupil. Antiphlogistic measures, mercury, and the use of belladonna, are the means to be employed. In acute cases we must bleed generally and locally, repeating the evacuation until the inflammation is subdued; give active purgatives, followed by saline aperients and the tartrite of antimony; restrict the patient to low diet; guard the eye from all injurious external influences, and keep the body at rest, as well as the affected organ. When the disorder is less violent, cupping or leeches will be sufficient.

"General depletion may be had recourse to with propriety, whenever there is feverishness, particularly if the pulse be full and strong. I must observe, however, that the absence of such symptoms does not contraindicate the practice. If the local complaint be serious, and threaten mischief to the organ, the treatment may properly begin with loss of blood from the arm, unless there should be objections in the particular circumstances of the case; in the progress of the affection we should not hesitate to repeat the depletion whenever the state of the part, or of the system, or both, call for it." (P. 170.)

*Local applications* cannot be of much service in so serious an affection of parts comparatively internal. Poppy fomentation will, perhaps, be the most soothing, but cold applications may be employed, if more agreeable to the patient. *Blisters* are improper in the active state of the inflammation.*

*Mercury*. The measures just mentioned lessen the violence of the inflammation, and the pain in the eye and head; but we frequently see, after large and repeated bleedings, that the action of the capillary vessels, the essential agents of the mischief, continues. Effusion of lymph goes on, and leads to alterations of structure. Some further power is necessary to put a stop to this disorganizing and destructive process, and that power is afforded by mercury, given in such a way as to produce quickly a decided effect upon the system. Small doses of mercury are of no avail. From two to four grains of calomel, with from one fourth to half a grain of opium, may be given every four, six, or eight hours, according to the urgency of the case. Under particular circumstances, bluepill, the hydr. c. creta, or mercurial frictions, may be employed instead of calomel. Two important questions here present themselves: first, to what extent mercury should be used; and secondly, how long should it be continued. The more powerful its action on the system, the more effectually does it control the disease.

"Sometimes these ends are not accomplished by a slight action on the mouth, when a more powerful influence will quickly do the business. Full salivation, quickly produced, cuts short recent disease, as if by a charm. The remedy may then be suspended, and its effects allowed to subside slowly, which will take two or three weeks: it will not be necessary to give any more mercury. Although the disease yields more quickly and effectually to a powerful mercurial action, it will be sufficient, in general, to make the remedy sensible in the mouth. In cases of longer standing its influence is not so quickly effectual. We must persevere until the lymph is absorbed, until the natural colour of the iris returns, the red zone round the cornea is gone, and vision is restored. This will require four, six, or eight weeks in some instances. A longer time is usually necessary in relapses and second attacks, than on the first occurrence of the complaint. I attended a gentleman for an attack of syphilitic iritis, in which the disease was of chronic character; it yielded slowly, and was well in six weeks. It recurred in consequence of premature exposure to cold, and the patient was obliged to keep the house for twelve weeks in a state of salivation, recovering perfect vision, which has continued unimpaired from that time, now many years ago." (P. 173.)

Mr. Lawrence observes, that iritis, of whatever kind, is

* Case 1.

easily managed, and that it rarely fails to yield to proper treatment, even when the case has been originally neglected. The occurrence of ptyalism is, in general, attended with decided improvement in all the symptoms.

"In comparing the progress, effects, and treatment of iritis with those of other inflammations, our attention is chiefly directed to the effusion which takes place from the inflamed texture, to its influence on the pupil, and to the paramount importance of stopping that effusion, and producing the absorption of the newly deposited substance. A very small quantity of lymph thrown out in the pupil, and then organized, may impair or destroy sight. A similar occurrence in any other part would be of no consequence. Effusions take place into the serous cavities, leading to the formation of adventitious membranes, or unnatural adhesions, without any subsequent injurious influence on the functions of the parts. Interstitial deposition occurs in other organs with similar results." (P. 178.)

Several cases are detailed, which exemplify the good effects of mercurial treatment, when active antiphlogistic means had been employed without success. Some place unlimited confidence in the power of mercury *alone,* while others discard it entirely, believing that other antiphlogistic means are capable of accomplishing all that is required. Mr. Lawrence cannot adopt either of these views.

"The practical conclusion at which I have arrived after ample experience of the complaint, under every variety of treatment, is, that iritis generally, and the syphilitic form of the complaint particularly, will be most advantageously treated by the successive or combined employment of antiphlogistic means and mercury; that this plan will give the quickest relief, will most effectually arrest the inflammation, restoring the iris to its healthy structure and functions, and will afford the best security against the return of the disease." (P. 181.)

Even where the apparently permanent effects of inflammation had been produced, as organised adhesions, and considerable imperfection of sight, mercury has often been of service, and has essentially improved vision. Mercury is useful in the idiopathic, as well as the syphilitic form of the disease.

"The local employment of mercury has been recommended in addition to its internal use. A weak solution of the oxymuriate has been used as a lotion; and the red precipitate ointment, also in a mild form, has been introduced between the lids. Such local stimuli are obviously inadmissible in the active stage of the inflammation; and I believe that they are of no use as mercurials, at any period. One mode of using the remedy locally is often of much service. When patients complain of severe pain over the orbit at night, the mercurial ointment combined with opium may be rubbed on the neighbouring integuments of the forehead and temple, with great alleviation of suffering. Six grains of the ointment with two grains of finely powdered opium should be well rubbed in before the time at which the nocturnal pain is expected to recur. By this mode of proceeding, for which we are indebted to the Germans, the attack of pain will generally be prevented. The benefit, however, is confined to the relief of this particular symptom: mercurial frictions on the brow do not arrest the inflammation as the internal use of the remedy does." (P. 182.)

*Artificial Dilatation of the Pupil.* The state of the pupil is one of the most important points in all cases of iritis: to preserve its circular figure, its natural dimensions, and permeability to light, is our principal object in most instances. Here we derive essential assistance from that anomalous and hitherto unexplained power which certain narcotic vegetables possess of dilating the pupil, as belladonna, hyoscyamus, laurus cerasus, and datura stramonium. Mr. L. prefers the solution of belladonna. Two or three drops (one scruple to one ounce of distilled water) should be introduced between the lids. The influence upon the iris is generally produced in half an hour, or from that to an hour, and the dilatation of the pupil lasts for several hours, or even some days. Mr. L. believes that the notion of belladonna being injurious to vision, is unfounded. The kind of amaurosis which is sometimes produced by its use goes off as the action of the iris returns, producing no permanent injury. Mr. L. has known instances in which it has been employed daily for many years, but it has merely dilated the pupil, without injuring sight or doing harm in any way. We learn, too, from such cases, the important fact that its influence on the iris is not, in the slightest degree, diminished by use.

"Belladonna and the other narcotics are capable of dilating the pupil in many instances, where the iris is no longer affected by variations in the quantity of light. But the permanent condensation of this delicate texture by the effusion of lymph under violent inflammation, when allowed to proceed uncontrolled, renders it altogether incapable of motion; consequently, in such cases, the narcotics have no effect on the pupil.

"The artificial dilatation of the pupil must be combined with the use of mercury in order to prevent that contraction to which there is so strong a tendency in iritis. Belladonna and the other narcotics do not exert their power when the iris is highly inflamed, and the disease not yet checked. The application, however, although it may fail to produce the desired effect, does no harm, especially if it be confined to the surrounding skin, and not actually dropped into the eye: perhaps it may be advantageous by preventing further contraction.

"The use of belladonna is of great importance, not only in preventing further diminution of the pupil, but because the contraction of the iris, under its powerful influence, is capable, where adhesions have already taken place, if the effusion be recent, of elongating them, and sometimes of separating them entirely, so as completely to liberate the pupillary margin. But the belladonna cannot do this alone: two conditions are necessary to the accomplishment of the object; the case must be recent, and with the employment of the belladonna we must produce, as quickly as possible, a full mercurial effect on the system. Under these circumstances, I have seen the whole edge of the pupil detached from the capsule of the lens, to which it had become adherent; and, in proof that adhesions had previously existed, the capsule has exhibited a circular arrangement of black spots, marking their number and situation. These marks, of which the dark colour, like that of the adhesions, is derived from the pigment of the uvea, are permanent, so far as my observation goes." (P. 191.)

Mr. Lawrence employs mercury in inflammation of the retina, whether acute or chronic, almost as confidently as in iritis. Mr. Hugh Carmichael, of Dublin, has lately recommended oil of turpentine in iritis generally, and particularly in the syphilitic form, in cases where mercury is inadmissible from its injurious influence, or from the debility produced by protracted disease: dose, a drachm three times a day, in almond emulsion. It is sometimes necessary to give the turpentine in larger quantities. Mr. Carmichael states that this remedy has seldom failed in iritis, although it has been extensively tried, and in very urgent cases. If the bowels are constipated, it appears that the beneficial effects of the turpentine are suspended. The cases related by Mr. Carmichael exhibit the powers of this remedy in a very favorable light. In several well-marked instances of syphilitic iritis, the pain, redness, and other symptoms, were quickly removed; effused lymph was soon absorbed, and vision restored under its influence. In other instances it was less successful. Mr. Guthrie reports that "in some cases it has succeeded admirably; in others it has been of little service, and in some unequal to the cure of the complaint." Mr. Lawrence has had no experience himself of the use of turpentine in iritis. Twenty-nine cases of syphilitic iritis are detailed.

The last chapter of the work is on *Syphilitic Ulceration of the Eyelids.*

